# Astrocytic CCAAT/Enhancer-Binding Protein Delta Contributes to Glial Scar Formation and Impairs Functional Recovery After Spinal Cord Injury

**DOI:** 10.1007/s12035-015-9486-6

**Published:** 2015-10-28

**Authors:** Shao-Ming Wang, Jung-Yu C. Hsu, Chiung-Yuan Ko, Nai-En Chiu, Wai-Ming Kan, Ming-Derg Lai, Ju-Ming Wang

**Affiliations:** 1 Institute of Basic Medical Sciences, College of Medicine, National Cheng Kung University, Tainan, 701 Taiwan; 2 Department of Cell Biology and Anatomy, College of Medicine, National Cheng Kung University, Tainan, 701 Taiwan; 3 Department of Pharmacology, College of Medicine, National Cheng Kung University, Tainan, 701 Taiwan; 4 Institute of Bioinformatics and Biosignal Transduction, College of Bioscience and Biotechnology, National Cheng Kung University, Tainan, 701 Taiwan; 5 Center of Molecular Inflammation, National Cheng Kung University, Tainan, 701 Taiwan; 6 Graduate Institute of Medical Sciences, Taipei Medical University, Taipei, 110 Taiwan; 7 Center for Neurotrauma and Neuroregeneration, Taipei Medical University, Taipei, 110 Taiwan; 8 Graduate Institute of Neural Regenerative Medicine, College of Medical Science and Technology, Taipei Medical University, Taipei, 110 Taiwan

**Keywords:** Astrogliosis, C/EBPδ, MMP-3, RhoA, Astrocyte migration

## Abstract

After spinal cord injury, inflammatory reaction induces the aggregation of astrocytes to form a glial scar that eventually blocks axonal regeneration. Transcription factor CCAAT/enhancer-binding protein delta (C/EBPδ) is a regulatory protein of genes responsive to inflammatory factors, but its role in glial scar formation after spinal cord injury remains unknown. By using a model of moderate spinal cord contusion injury at the mid-thoracic level, we found that C/EBPδ was expressed mostly in the reactive astrocytes bordering the lesion in wild-type mice from 7 days after the injury. *C*/*EBPδ*-deficient mice showed reduced glial scar formation, more residual white matter, and better motor function recovery compared with wild-type mice 28 days after the injury. Upon interleukin (IL)-1β stimulation in vitro, the increased expression of C/EBPδ in reactive astrocytes inhibited *RhoA* expression and, subsequently, the ability of astrocyte migration. However, these reactive astrocytes also produced an increased amount of matrix metalloproteinase-3, which promoted the migration of non-IL-1β-treated, inactive astrocytes. Although the involvement of other non-astroglial C/EBPδ cannot be entirely excluded, our studies suggest that astrocytic C/EBPδ is integral to the inflammatory cascades leading to glial scar formation after spinal cord injury.

**Electronic supplementary material:**

The online version of this article (doi:10.1007/s12035-015-9486-6) contains supplementary material, which is available to authorized users.

## Introduction

After spinal cord injury (SCI), astrocytes in the lesion site become reactive and aggregate to form a glial scar, which is one of the major obstacles to successful axonal regeneration. The glial scar not only imposes a physical barrier to regenerative axons but also produces inhibitory molecules, such as chondroitin sulfate proteoglycans, that chemically block the regrowth of injured axons across the lesion [1, 2], leading to the failure of functional recovery [3]. Despite its detrimental role in the injured spinal cord, the glial scar may benefit wound healing by preventing inflammatory cells and harmful substances in the lesion core from spreading out, thereby protecting the originally uninjured tissue from secondary injuries [4, 5]. Therefore, more in-depth understanding of reactive astrocytes and glial scar formation may improve future therapeutic possibilities for SCI.

Glial scar formation results primarily from enhanced migration of reactive astrocytes toward the lesion site [6–9] with a smaller contribution from the proliferation of migrating or resident astrocytes [10–12]. Astrocyte motility relies largely on the integrity of intermediate filaments [13] and the dynamics of the actin cytoskeleton mediated by the Rho family of small GTPases, including Rho and Rac1 [12, 14]. In addition, matrix metalloproteinases (MMPs) also contribute to astrocyte migration by proteolytic remodeling of extracellular matrix molecules [12, 15–17].

After SCI, disrupted blood vessels in the lesion allow the infiltration of inflammatory cells and the release of cytokines, including interleukins (ILs), transforming growth factor-β, and interferon-γ [18–20]; this not only exacerbates the extent of the primary injury but also induces astrocyte reactivity and glial scar formation [1, 21]. Thus, inflammatory processes following the injury are critical to secondary pathogenesis, including glial scarring.

Transcription factor CCAAT/enhancer-binding protein delta (C/EBPδ) belongs to the CCAAT/enhancer-binding protein (C/EBP) family. This protein is expressed at relatively low levels under normal physiological conditions and is upregulated in a number of inflammatory diseases by a variety of extracellular stimuli, such as IL-6, IL-1β, and tumor necrosis factor (TNF)-α [22–24]. Activated C/EBPδ in astrocytes promotes chemo-attraction and migration of microglia/macrophages [24]; it also contributes to the resistance of cell death [24] and attenuates macrophage-mediated phagocytosis of damaged neurons [23], suggesting its involvement in neuro-inflammatory and anti-apoptotic responses.

Inflammatory cytokine IL-1β not only activates C/EBPδ expression but also reduces astrocyte migration via de-activation of the Rho/Rock signaling axis [25]. Nevertheless, whether and how C/EBPδ regulates the transcription of *Rho* and *Rock* genes in astrocytes has not been investigated. Moreover, phosphorylation of C/EBPδ at Ser167 in astrocytes is associated with the transcription of genes encoding MMP-1 and MMP-3, which are implicated in macrophage/microglia migration [26]. However, the role of C/EBPδ in modulating astrocyte motility and glial scar formation after SCI remains unknown.

In this study, we hypothesized that C/EBPδ plays a regulatory role in the inflammatory responses that follow SCI and, therefore, contributes to glial scar formation in the injured spinal cord. To test this hypothesis, we compared a number of wound healing events, including glial scarring, white matter sparing, and motor function recovery, between wild-type and *C*/*EBPδ*-deficient (*C*/*EBPδ* ^−/−^) mice after SCI. We also investigated how C/EBPδ affected the migratory behavior of cultured astrocytes in vitro. Our results provide new insights into the functional role of C/EBPδ in glial scar formation and may lead to novel therapeutic strategies for the treatment of SCI.

## Results

### C/EBPδ Expression in Astrocytes Is Associated with Glial Scar Formation After SCI

**Fig. 1:**
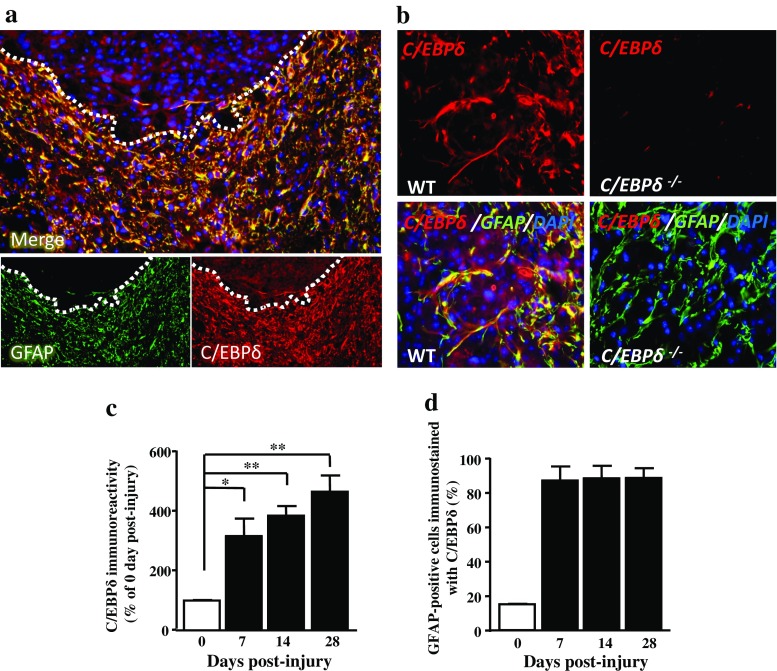
C/EBPδ is associated with GFAP-positive astrocytes in the glial scar of wild-type mice after SCI. **a** Transverse sections of the spinal cord obtained from wild-type mice are immunostained with anti-GFAP and -C/EBPδ antibodies at 14 days after spinal cord injury. Both GFAP and C/EBPδ immunoreactivity is apparent in the residual cord tissue (*dotted lines*) ventrolateral to the lesion epicenter particularly along the lesion border. **b** At higher magnification, co-localization of GFAP and C/EBPδ immunoreactivity is evident in astrocytes of wild-type mice, whereas C/EBPδ immunoreactivity is negative in astrocytes of C/EBPδ^−/−^ mice. **c** Quantitative analyses (*n* = 6 for each time point) show that the intensity of C/EBPδ immunoreactivity increases significantly over time in the injured spinal cord. **d** The percentage of GFAP-positive astrocytes that is co-localized with C/EBPδ immunoreactivity increases from 7 days post-injury onwards

To investigate the role of C/EBPδ in glial scar formation, we first examined the expression of C/EBPδ in the injured spinal cord of wild-type mice. After contusive SCI, glial fibrillary acidic protein (GFAP)-positive astrocytes formed a glial scar surrounding the lesion epicenter. Co-localization of GFAP and C/EBPδ immunoreactivity was evident along the lesion border (Fig. 1a and Sup. Fig. 1). To further confirm its specificity, the anti-C/EBPδ antibody was applied to sections of *C*/*EBPδ* ^−/−^ mice as the negative control, and no positive immunostaining was found (Fig. 1b). Quantitative analysis showed that the expression of C/EBPδ increased in the injured spinal cord over time (Fig. 1c) and was strongly associated with GFAP-positive astrocytes in the vicinity of the lesion epicenter, particularly in the glial scar (Fig. 1d). These findings suggest that the expression of C/EBPδ in astrocytes participates in glial scar formation after SCI in mice.

### *C*/*EBPδ* Deficiency Improves Motor Function Recovery After SCI

**Fig. 2:**
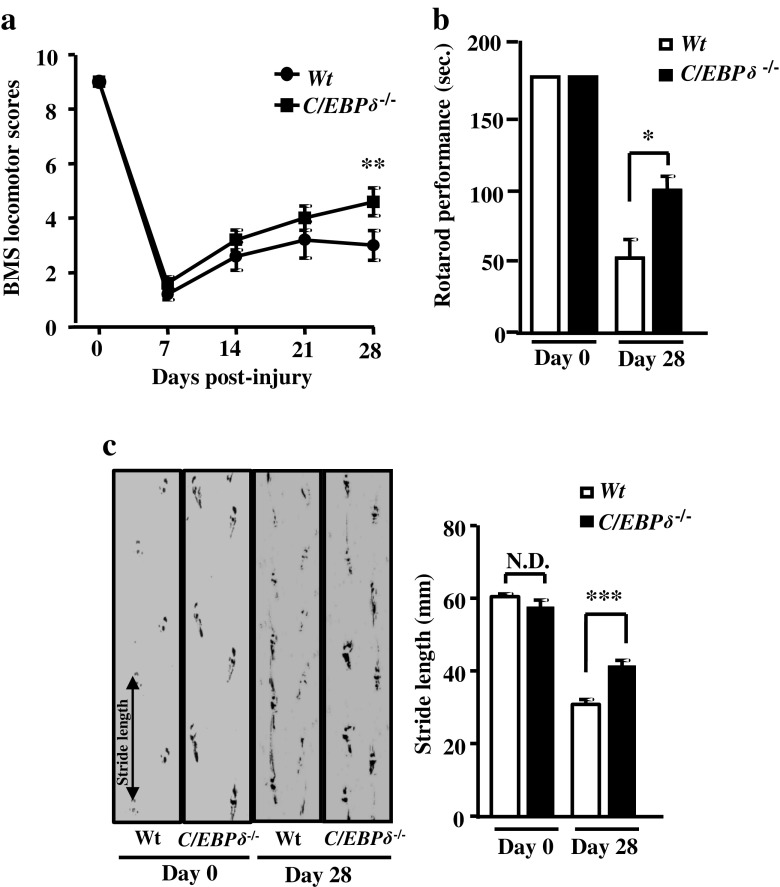
*C*/*EBPδ* ^−/−^ mice exhibit improved functional recovery compared with wild-type mice after SCI. **a** Based on the 9-point BMS locomotor rating scale, *C*/*EBPδ* ^−/−^ mice show better motor function recovery in the open field than the wild-type mice after SCI, particularly at 28 days after injury (*n* = 6 per genotype). **b** Likewise, *C*/*EBPδ* ^−/−^ mice perform significantly better on a rotarod. **c** *C*/*EBPδ* ^−/−^ mice show longer stride length on the footprint analysis than the wild-type mice 28 days after SCI (*n* = 6 per genotype)

To evaluate the effects of C/EBPδ on the recovery of motor behavior after SCI, functional improvements were assessed by a battery of behavioral tests, including open-field locomotion, performance on a rotarod, and footprint analyses in wild-type and *C*/*EBPδ* ^−/−^ mice. Mice of both genotypes showed comparable functional outcomes in all three behavioral tests before SCI (day 0; Fig. 2a–c). On day 28 after the injury, however, *C*/*EBPδ* ^−/−^ mice exhibited significantly higher BMS locomotor scores, better rotarod performance, and longer stride length of the hindlimb compared with the wild-type mice (Fig. 2a–c). Our results indicate that C/EBPδ is associated with functional disabilities and that genetic deletion of *C*/*EBPδ* promotes the recovery of hindlimb motor function after SCI.

### *C*/*EBPδ* Deficiency Results in Decreased Glial Scar Formation and Increased White Matter Sparing After SCI

**Fig. 3:**
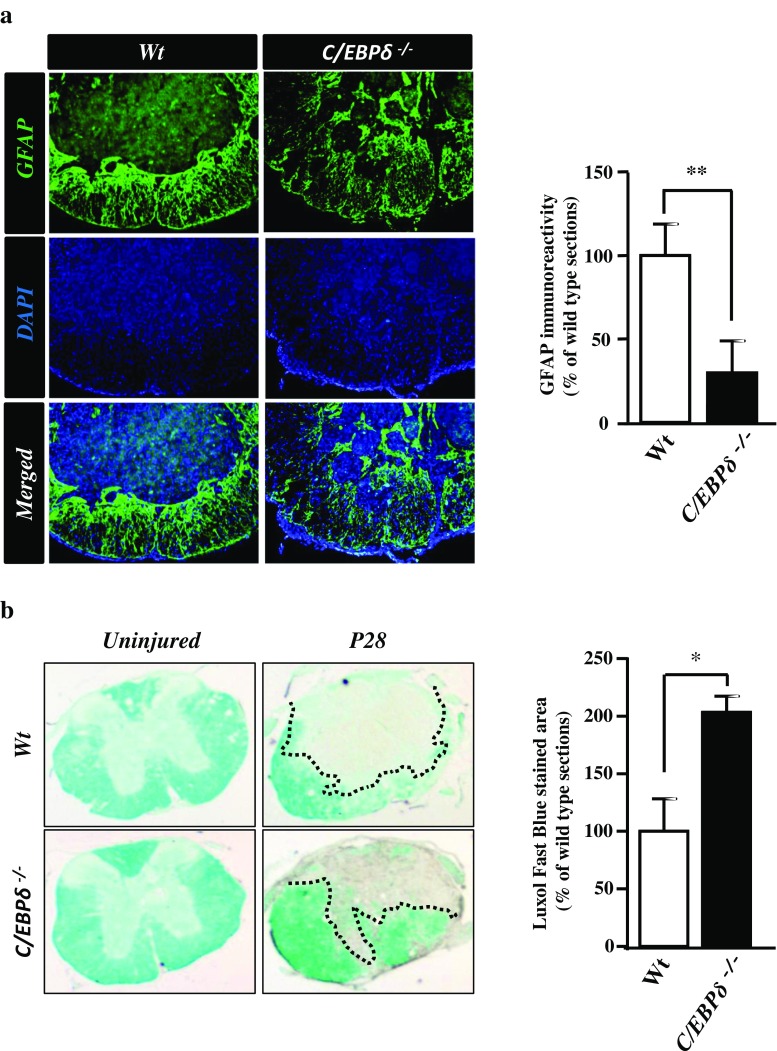
*C*/*EBPδ* ^−/−^ mice display less severe glial scar formation and more residual white matter than wild-type mice after SCI. **a** Transverse sections of the spinal cord are immunostained with anti-GFAP antibody 28 days after SCI. GFAP-positive astrocytes of the *C*/*EBPδ* ^−/−^ mice are relatively dispersed, loosely aggregated in the glial limitans where the glial scar is formed compared with those of the wild-type mice, suggesting a fragmental, less severe glial scar in the *C*/*EBPδ* ^−/−^ mice. Statistical analysis reveals that the intensity of GFAP immunoreactivity is lower in the *C*/*EBPδ* ^−/−^ mice than in the wild-type mice (*n* = 6 per genotype). **b** Transverse sections of the spinal cord are stained with Luxol Fast Blue to visualize the residual white matter around the lesion epicenter 28 days after SCI. *C*/*EBPδ* ^−/−^ mice show more prominent Luxol Fast Blue staining and quantitatively larger area of residual white matter than the wild-type mice (*n* = 6 per genotype). *Dotted lines* demarcate the residual cord tissue

In the injured spinal cord, the glial scar mainly consists of reactive astrocytes and is a major barrier that blocks neurite extension and axonal regeneration during the chronic stage of SCI [27]. Our previous study showed that C/EBPδ contributes to astrogliosis in Alzheimer’s disease [26]. The glial scar is formed with substantially increased expression of astrocytic GFAP. In the present study, we found that GFAP immunostaining was more widespread in the *C*/*EBPδ* ^−/−^ mice 28 days after SCI but the area with intense GFAP immunoreactivity was loosely distributed and fragmental around the lesion compared with that in the wild-type mice (Fig. 3a). This finding suggests a milder and less severe glial scar in *C*/*EBPδ* ^−/−^ mice than in the wild-type mice after SCI. To determine whether *C*/*EBPδ* deficiency and the consequent less severe astrogliosis affect the sparing of cord tissue after the injury, we further quantified the size of residual white matter around the lesion epicenter in *C*/*EBPδ* ^−/−^ and wild-type mice using Luxol Fast Blue staining. Residual white matter has been demonstrated to be the best single measurement for the severity of injury in the contused spinal cord and is predictive of motor function recovery [28]. We found that the area of residual white matter was significantly larger in *C*/*EBPδ* ^−/−^ mice than in wild-type mice 28 days after SCI (Fig. 3b). This may account for the wider area of GFAP immunostaining observed above in the *C*/*EBPδ* ^−/−^ mice (Fig. 3a). These data suggest that C/EBPδ is involved in glial scar formation and depletion of C/EBPδ not only reduces glial scarring but also promotes white matter sparing after SCI.

### C/EBPδ Does not Affect Astrocyte Proliferation but Impedes Astrocyte Migration

**Fig. 4:**
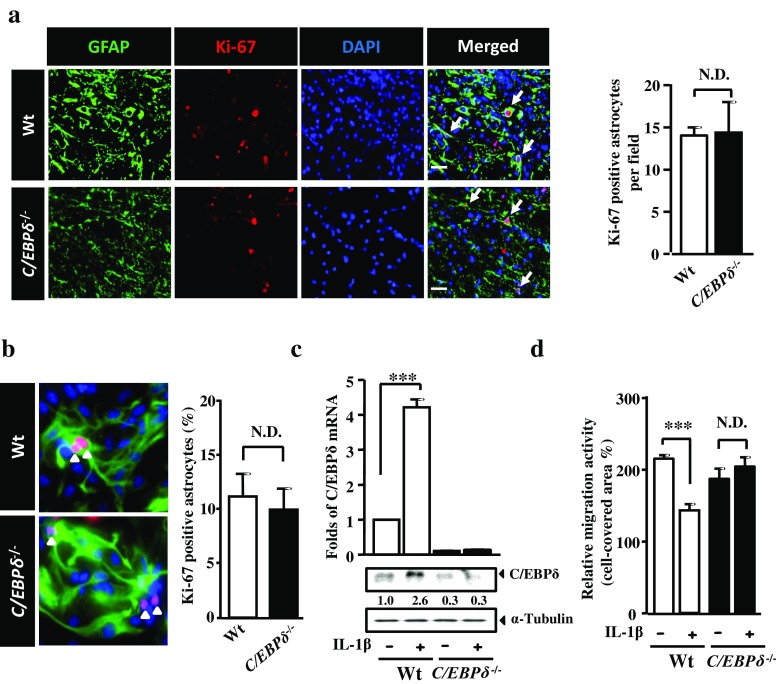
C/EBPδ does not promote astrocytic proliferation but does affect migration. **a** Transverse sections of the spinal cord are immunostained with anti-GFAP antibody to label reactive astrocytes and the anti-Ki-67 antibody to identify proliferative cells. Quantitative analysis shows that the number of proliferative astrocytes is comparable between *C*/*EBPδ* ^−/−^ and wild-type mice 7 days after SCI. **b** Similarly, cultured primary astrocytes isolated from the cortex of *C*/*EBPδ* ^−/−^ and wild-type mice exhibit no statistical difference in cell proliferation, suggesting that C/EBPδ does not affect astrocyte proliferation. **c** qPCR and Western blots reveal that the expression of both C/EBPδ mRNA and protein increases significantly in wild-type astrocyte cultures treated with IL-1β, an inflammatory cytokine known to activate C/EBPδ expression, for 3 h compared with all the other groups. **d** A scratch wound assay is conducted with IL-1β treatment using primary cultures of *C*/*EBPδ* ^−/−^ and wild-type astrocytes as described in the “Materials and Methods” section. Wild-type astrocytes treated with IL-1β manifest reduced migration compared with untreated wild-type astrocytes. However, the migration of *C*/*EBPδ* ^−/−^ astrocytes is not affected regardless of IL-1β treatment. Data obtained from three triplicates are analyzed. *Arrows* and *arrowheads* point to proliferative astrocytes

We have previously demonstrated that glial scar formation is primarily attributed to astrocyte migration toward the lesion with a relatively minor contribution from astrocyte proliferation [12]. Nevertheless, a recent study shows that the glial scar immediately borders the lesion core is formed by newly proliferated astrocytes with elongated morphology [29]. We thus investigated the involvement of C/EBPδ in both astrocyte migration and proliferation using immunofluorescence in vivo and in vitro. Our quantitative results showed that the number of GFAP-positive astrocytes double-labeled with Ki-67, a cell proliferation marker, were comparable between wild-type and *C*/*EBPδ* ^−/−^ mice 7 days after SCI (Fig. 4a). With the use of primary cultures of astrocytes purified from wild-type or *C*/*EBPδ* ^−/−^ mice, similarly, there was no statistically significant difference in the number of proliferative astrocytes immunolabeled with anti-GFAP and -Ki-67 antibodies (Fig. 4b). These results suggest that *C*/*EBPδ* deficiency has no detrimental effect on astrocyte proliferation. Furthermore, we examined the contribution of C/EBPδ in astrocyte migration using a scratch wound paradigm in vitro with inflammatory cytokine IL-1β to stimulate astrocyte reactivity. IL-1β is known to activate the expression of C/EBPδ in human glioblastoma-astrocytoma U373MG cells and is expressed abundantly in the injured spinal cord [18]. Here, we found that IL-1β upregulated both the levels of C/EBPδ mRNA and protein in cultured wild-type astrocytes in vitro (Fig. 4c). Interestingly, however, the migration of wild-type astrocytes was significantly attenuated with IL-1β treatment, whereas the migratory behavior of *C*/*EBPδ* ^−/−^ astrocytes was not affected (Fig. 4d). These findings indicate that the expression of C/EBPδ mRNA and/or protein reduces the migration of IL-1β-treated wild-type astrocytes in vitro.

### C/EBPδ Attenuates Astrocyte Self-Migration Through the Inhibition of RhoA

**Fig. 5:**
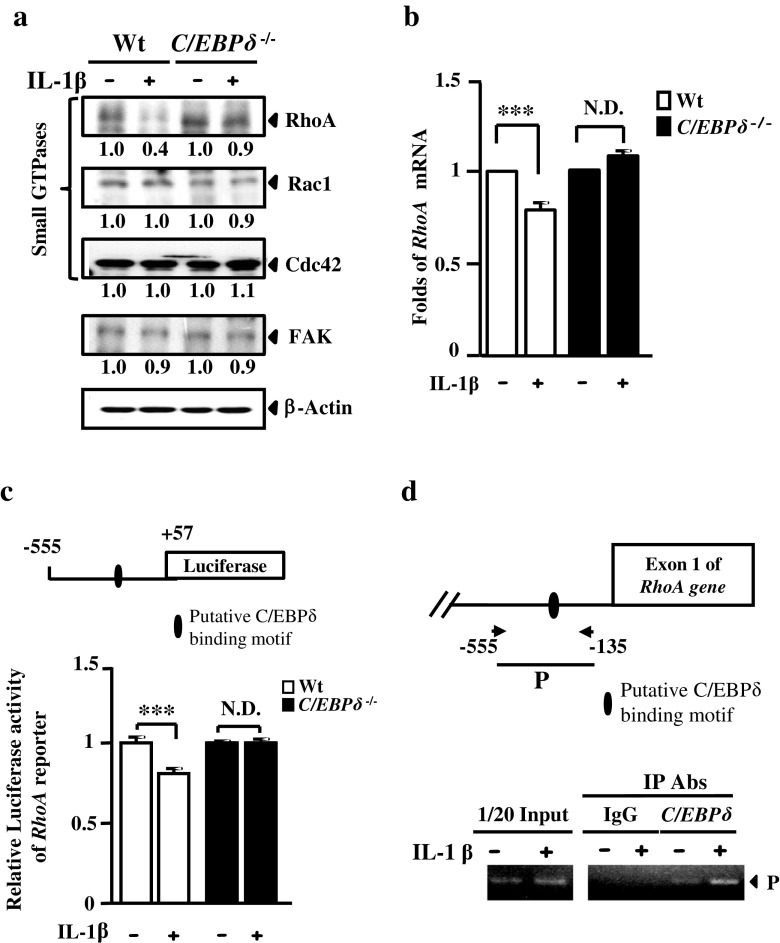
C/EBPδ represses RhoA expression and contributes to attenuated migration of IL-1β-treated astrocytes. **a** Protein lysates harvested from IL-1β-treated *C*/*EBPδ* ^−/−^ or wild-type astrocytes are examined by Western blots to detect the expression of small GTPases RhoA, Rac1, and Cdc42. Densitometrical data obtained from three triplicates are quantified. The results are normalized with β-actin and presented as the change in folds relative to the IL-1β-untreated wild-type control. The expression of RhoA is substantially reduced in wild-type astrocytes treated with IL-1β for 6 h compared with astrocytes of the other condition or genotype, although the expressions of Rac1, Cdc42, and FAK are not affected. **b** qPCR also confirms a significant reduction in *RhoA* mRNA in wild-type astrocytes treated with IL-1β for 3 h. **c** A reporter assay demonstrates similar outcomes using primary astrocytes transfected with the *RhoA* reporter, followed by IL-1β treatment and detection of the luciferase activity. **d** A ChIP assay is conducted using IL-1β-treated primary wild-type astrocytes. Immunoprecipitated product (P) captured by anti-C/EBPδ antibody contains C/EBPδ and its binding target RhoA promoter, which is subsequently verified by PCR. The results suggest that C/EBPδ represses *RhoA* transcription by direct binding to *RhoA* promoter in IL-1β-treated wild-type astrocytes

The expression of several key regulators, such as RhoA, Rac1, Cdc42, and FAK, are involved in the signaling pathways that promote cell migration [30–32]. Moreover, a previous study has demonstrated that IL-1β induces reactive astrogliosis by de-activating a signaling pathway mediated by Rho GTPase and its downstream effector Rho kinase (ROCK) in human astrocytes [25]. To elucidate the role of C/EBPδ in attenuated astrocyte migration, we examined the expression of RhoA, Rac1, Cdc42, and FAK in wild-type and *C*/*EBPδ* ^−/−^ astrocytes treated with or without IL-1β in vitro. The result showed that RhoA, but not Rac1, Cdc42, or FAK, was specifically and substantially inhibited in IL-1β-treated wild-type astrocytes, whereas such a reduction in RhoA was not observed in IL-1β-treated *C*/*EBPδ* ^−/−^ astrocytes (Fig. 5a). Moreover, we conducted quantitative PCR (qPCR) to assess whether C/EBPδ attenuated *RhoA* transcription at the mRNA level. The result showed that, with IL-1β treatment, *RhoA* transcription was significantly reduced in wild-type astrocytes but remained unaffected in *C*/*EBPδ* ^−/−^ astrocytes (Fig. 5b). In a reporter assay, furthermore, we found that the activity of the *RhoA* reporter was inhibited in IL-1β-treated wild-type astrocytes but was unchanged in *C*/*EBPδ* ^−/−^ astrocytes treated with/without IL-1β (Fig. 5c). We also conducted a chromatin immunoprecipitation (ChIP) DNA binding assay followed by PCR in vivo to further verify whether C/EBPδ repressed *RhoA* transcription by directly binding to its promoter. The results showed a direct binding of C/EBPδ to the promoter of *RhoA* in IL-1β-treated wild-type astrocytes, evidenced by immunoprecipitation of cross-linked C/EBPδ and its target RhoA promoter, as well as subsequent PCR outcomes (Fig. 5d). These results suggest that attenuated migration of astrocytes that express C/EBPδ is a consequence of RhoA inhibition.

**Fig. 6:**
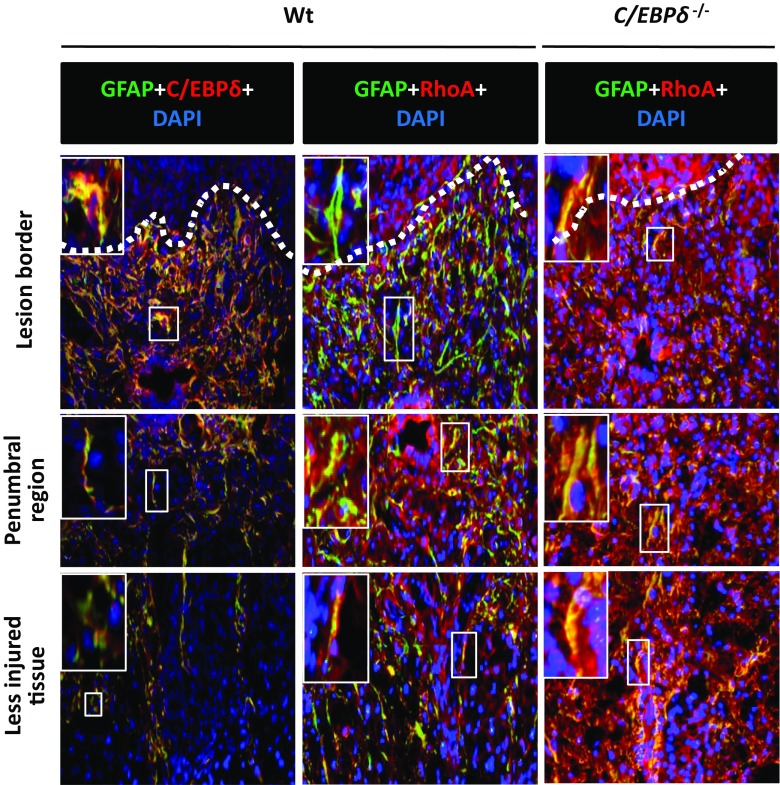
The expression of *RhoA* is attenuated in C/EBPδ-positive astrocytes along the glial limitans in the immediate vicinity of the lesion epicenter 14 days after SCI. Immunostaining demonstrates a regional heterogeneity in the expression of C/EBPδ and RhoA in injured wild-type mice. GFAP-positive astrocytes boarding the lesion epicenter (*dotted line*) show increased C/EBPδ but reduced RhoA expression, whereas those at the penumbral region between the lesion border and the less injured tissue exhibit converse results. In the *C*/*EBPδ* ^−/−^ mice, RhoA is expressed in astrocytes ubiquitously in the injured spinal cord. The *boxed area* is enlarged in the upper right corner of each photograph

To further determine the correlation between astrocytic C/EBPδ and RhoA, we used double immunostaining to locate the expression of C/EBPδ and RhoA in the injured spinal cord 14 days after SCI. In the wild-type mice, strong C/EBPδ immunoreactivity was found in reactive astrocytes located in the glial scar along the lesion border where the intensity of RhoA immunoreactivity was low. Conversely, however, astrocytes in the penumbral region between the lesion border and the less injured tissue exhibited weak C/EBPδ immunoreactivity but relatively high RhoA expression (Fig. 6 and Sup. Fig. 2). Injured *C*/*EBPδ* ^−/−^ mice, on the other hand, showed consistent RhoA immunoreactivity in astrocytes anywhere in the residual cord tissue (Fig. 6). This finding suggests that C/EBPδ inhibits RhoA expression, especially in reactive astrocytes located in the glial scar at the edge of the lesion epicenter after SCI.

### C/EBPδ Induces Astrocytic Expression of MMP-3, Which Promotes the Migration of Inactive Astrocytes

Although our in vitro outcomes demonstrated that C/EBPδ inhibited astrocytic expression of RhoA and thus astrocyte migration (Fig. 5), this finding contradicted our in vivo study that wild-type mice showed more intense astrogliosis abutting the lesion compared with *C*/*EBPδ* ^−/−^ mice after SCI (Fig. 3). This discrepancy implied a possibility that the inhibitory effect of C/EBPδ on RhoA and consequent inhibition of astrocyte migration was counteracted by other predominant promoting factors, giving rise to enhanced astrocyte motility and astrogliosis in injured wild-type mice in vivo.

**Fig. 7:**
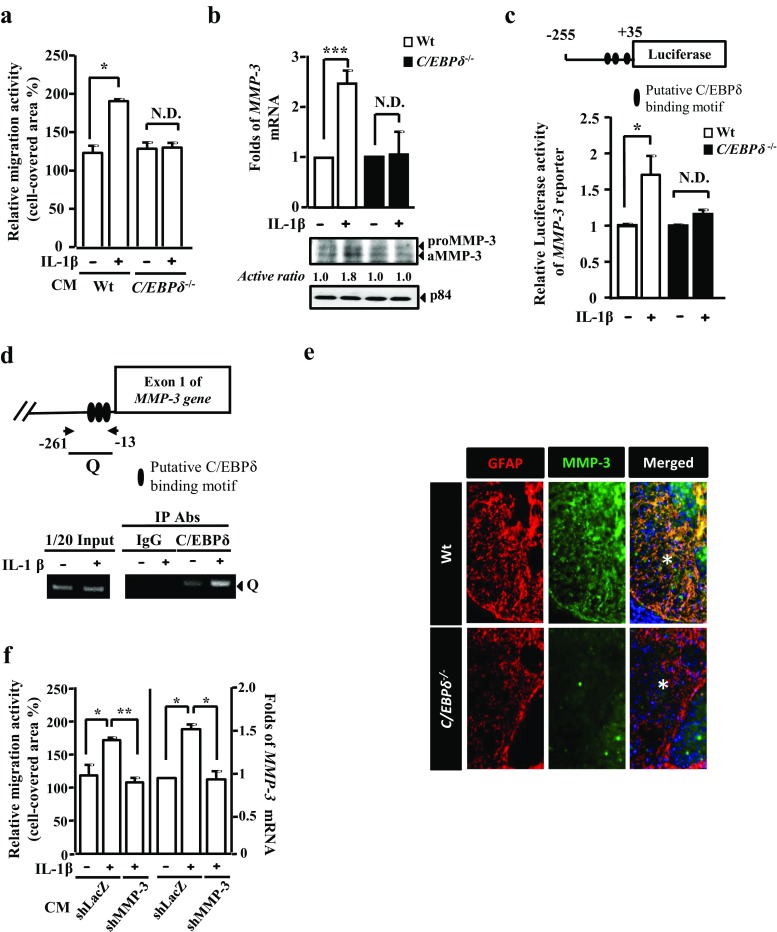
Conditioned medium from wild-type astrocytes expressing C/EBPδ promotes migration of the inactive astrocytes through MMP-3 activation. **a** In a wound healing assay, IL-1β-untreated, inactive wild-type astrocytes grow in conditioned media obtained from wild-type or *C*/*EBPδ* ^−/−^ astrocytes pretreated with or without IL-1β. Astrocytes show enhanced migration when cultured in the conditioned medium collected from IL-1β-pretreated wild-type astrocytes, suggesting a promoting effect of this conditioned medium on migratory behavior. **b** qPCR and Western blots reveal that the expression of both MMP-3 mRNA and protein increases significantly in wild-type astrocytes treated with IL-1β for 6 h. p84 is used as the loading control for Western blots. **c** A reporter assay shows that, when wild-type or *C*/*EBPδ* ^−/−^ astrocytes are transfected with the *MMP*-*3* reporter, luciferase activity increases significantly in IL-1β-treated wild-type astrocytes. **d** A ChIP assay demonstrates that, in IL-1β-treated wild-type astrocytes, immunoprecipitated product (Q) captured by anti-C/EBPδ antibody contains both C/EBPδ and its binding target on the *MMP*-*3* promoter, which is evidenced by the PCR, suggesting a direct binding of C/EBPδ to the *MMP*-*3* promoter. **e** Transverse sections of the spinal cord immunostained with anti-GFAP and -MMP-3 antibodies demonstrate that wild-type mice exhibit higher MMP-3 immunoreactivity in the penumbral region (*asterisks*) lying between the lesion epicenter and the less injured tissue compared with the *C*/*EBPδ* ^−/−^ mice 28 days after SCI. **f** A scratch wound assay of wild-type astrocytes shows that the conditioned medium harvested from IL-1β-treated shLacZ-knockdown astrocytes significantly enhances astrocyte migration, which can be attenuated by the conditioned medium collected from IL-1β-treated shMMP-3-knockdown astrocytes (*three left columns*). Altered migratory behavior of these astrocytes is consistently associated with their expression of MMP-3 mRNA (*three right columns*)

To explore this possibility, we first determined whether or not wild-type astrocytes, which expressed C/EBPδ upon IL-1β pretreatment, produced any promoting factors that facilitated astrocyte motility in vitro. Cultured wild-type and *C*/*EBPδ* ^−/−^ astrocytes were treated with or without IL-1β for 6 h, followed by a thorough rinse with phosphate-buffered saline (PBS) and replacement of culture media by serum-free Dulbecco’s modified Eagle’s medium (DMEM) for 12 h. Then, these four conditioned media were applied respectively to four groups of non-IL-1β-stimulated, inactive wild-type astrocyte cultures. With the use of scratch wound assays, we found a significant increase in astrocyte migration in the group cultured with the conditioned medium collected from IL-1β-pretreated wild-type astrocytes, compared with the other three groups (Fig. 7a); this suggests that astrocytes expressing C/EBPδ upon IL-1β stimulation indeed secrete factors that promote the migration of inactive astrocytes.

To further identify the promoting factors produced by astrocytes after IL-1β-induced C/EBPδ activation, a number of assays were conducted to analyze astrocytic expression of MMP-3 at protein and mRNA levels. Previously, we demonstrated an increased expression of *MMP*-*3* mRNA in response to C/EBPδ activation in glioblastoma-astrocytoma U373MG cells [26]. Here, we found that the quantity of MMP-3 mRNA and protein increased significantly after IL-1β treatment in primary cultures of wild-type astrocytes compared with those in *C*/*EBPδ* ^−/−^ astrocytes (Fig. 7b). Moreover, after three putative C/EBP binding motifs were identified (Fig. 7c, upper panel) using the EnsMart System (www.ensembl.org/biomart/martview/c6c926d9815de045873590a6da1ac151), our reporter assay demonstrated that the activity of the *MMP*-*3* reporter rose significantly after IL-1β treatment in primary cultures of wild-type astrocytes compared with that in *C*/*EBPδ* ^−/−^ astrocytes (Fig. 7c, bottom panel).

By using a ChIP DNA binding assay followed by PCR, furthermore, we found that C/EBPδ bound directly to the promoter region of the *MMP*-*3* gene in IL-1β-treated wild-type astrocytes in vitro (Fig. 7d). This finding was consistent with the results of immunofluorescence in vivo, which demonstrated higher MMP-3 immunoreactivity in GFAP-positive astrocytes around the lesion epicenter in the wild-type mice than in the *C*/*EBPδ* ^−/−^ mice 28 days after the injury (Fig. 7e).

In addition, a scratch wound assay was performed to verify the contribution of MMP-3 in the migration of inactive astrocytes. ShLacZ-knockdown and shMMP-3-knockdown astrocytes were treated with IL-1β, rinsed with PBS, and then incubated with serum-free DMEM for 12 h. Subsequently, the conditioned media were collected and applied respectively to primary cultures of IL-1β-untreated, inactive wild-type astrocytes for the scratch assay. The results showed that the conditioned medium collected from IL-1β-treated shLacZ-knockdown astrocytes significantly enhanced the migration of inactive astrocytes compared with that harvested from IL-1β-treated shMMP-3-knockdown astrocytes (Fig. 7f). This result suggests that IL-1β-stimulated astrocytes secret MMP-3, which in turn facilitates the migration of non-IL-1β-stimulated, inactive astrocytes.

### C/EBPδ is Integral to Glial Scar Formation After SCI

**Fig. 8:**
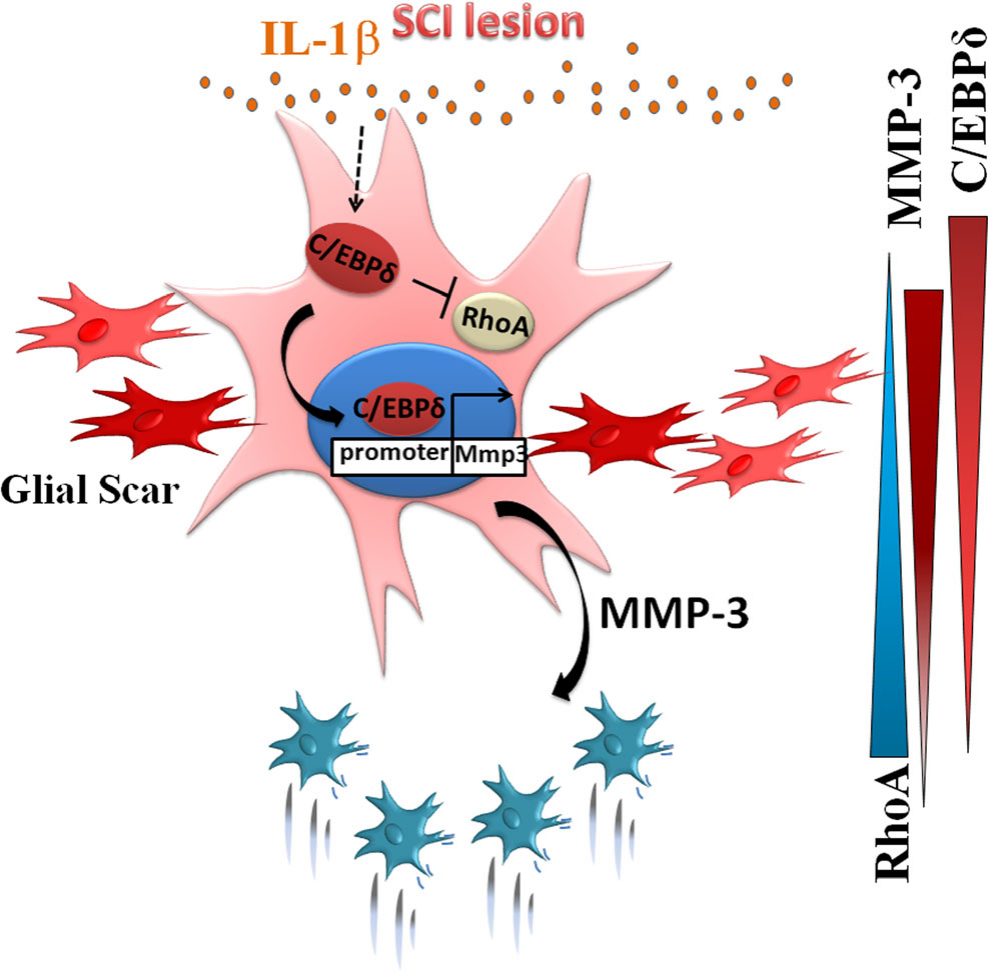
A schematic diagram illustrates the proposed model for the modulatory effect of C/EBPδ on astrocyte migration and glial scar formation after SCI. In response to the injury, inflammatory cytokine IL-1β released in the lesion epicenter activates astrocytic expression of C/EBPδ, which in turn inhibits RhoA expression and thus impedes the migration of the astrocytes located along the lesion border. Simultaneously, C/EBPδ upregulates astrocytic expression of MMP-3. Diffusion of MMP-3 promotes the migration of neighboring astrocytes, particularly those in the penumbral region between the lesion border and less injured cord tissue. Consequently, astrocytes in the penumbral region with relatively high RhoA expression and an enriched MMP-3 environment migrate toward the lesion border where they aggregate to form a glial scar. Relative gradients of C/EBPδ, RhoA, and MMP-3 are illustrated on the *right*

Taking all our findings together, we herein propose a model depicting how C/EBPδ modulates glial scar formation after SCI (Fig. 8). After SCI, inflammatory cytokine IL-1β released from the lesion epicenter activates the expression of C/EBPδ in reactive astrocytes located immediately along the lesion border. Activated C/EBPδ inhibits the cytoskeleton regulatory protein RhoA and consequently impedes the migration of these reactive astrocytes. Meanwhile, C/EBPδ in reactive astrocytes also enhances the expression of MMP-3, which predominantly promotes the migration of adjacent inactive astrocytes in the penumbral region toward the lesion border, giving rise to a densely packed glial scar.

## Discussion

Our studies provide novel evidence demonstrating that C/EBPδ promotes glial scar formation and plays dual roles in astrocyte migration after SCI. We show that C/EBPδ was expressed by reactive astrocytes along the lesion border and that *C*/*EBPδ* ^−/−^ mice exhibited reduced glial scar, more residual white matter, and better motor function recovery than wild-type mice after the injury. Although increased expression of C/EBPδ in response to IL-1β stimulation repressed *RhoA* transcription and thus inhibited astrocyte motility in vitro, C/EBPδ also enhanced the transcription and secretion of MMP-3, which selectively promoted the migration of non-IL-1β-treated, inactive astrocytes. Together, our results suggest that C/EBPδ can modulate astrocyte motility and is integral to glial scar formation after SCI.

Our study revealed that approximately 84 ∼ 90 % of C/EBPδ immunoreactivity co-existed with GFAP after SCI, indicating that non-astroglial cell types likely contributed to the other 10 ∼ 16 % of C/EBPδ expression. Similarly, our previous study shows that 95 % of C/EBPδ signal is co-localized with GFAP-positive cells in *App*Tg mice [26]. Moreover, C/EBPδ is expressed in both astrocytes and microglia in the brain of mice treated with lipopolysaccharide (LPS) [33]. Although the possible involvement of non-astroglial cells in the expression of C/EBPδ cannot be entirely excluded, astrocytic C/EBPδ appears to play a major role in glial scar formation during wound healing because C/EBPδ is expressed mostly by reactive astrocytes after SCI.

Our result of immunofluorescence showed that C/EBPδ was expressed in both nuclei and cytoplasm of astrocytes in the glial scar using the antibody previously described (Geneka Biotechnology, Montreal, Canada) [34]. Cytosolic C/EBPδ does not seem consistent with its role as a transcription factor. Interestingly, however, a previous study shows that cAMP-dependent protein kinase contributes to C/EBPδ shuttling from cytosol to the nucleus in osteoblasts [35], suggesting that cytosolic C/EBPδ exists. Nevertheless, the biological function of cytosolic C/EBPδ needs to be further investigated.

Astrogliosis has been observed under inflammatory conditions in several acute and chronic neurological disorders, including SCI, Alzheimer’s disease, and Parkinson’s disease [24, 36, 37]. Reactive astrogliosis is characterized by a densely packed, trabecular meshwork of astrocytes, which eventually forms a scar [37]. Glial scar formation is a complex process that involves the migration and proliferation of reactive astrocytes [12, 29]. IL-1β plays a specific role in the primary initiation of astrocytic reactivity in response to an injury [18]. Nevertheless, a previous study showed that IL-1β inhibits astrocyte migration through de-activation of the Rho/Rock1 pathway, which regulates the dynamics of the actin cytoskeleton [25, 38] and is associated with astrocyte motility in vitro and astrogliosis after SCI [39–41]. Similarly, RhoA knockdown inhibits the migration of cancer cells [42]. Consistent with those finding, we found that IL-1β upregulated astrocytic C/EBPδ, which in turn suppressed RhoA expression and the self-migration of the astrocytes in vitro. This effect of impeded self-migration was lost in IL-1β-treated *C*/*EBPδ* ^−/−^ astrocytes, suggesting an inhibitory role of C/EBPδ in RhoA expression and likely the downstream Rho/Rock signaling pathway, culminating in restrained astrocyte motility upon IL-1β stimulation.

Some research suggests that glial scar formation is beneficial to wound healing by preventing inflammatory cells, such as macrophages and microglia, in the lesion from infiltrating into the surrounding neural parenchyma to cause secondary damage [2, 4, 5, 43, 44]. In our immunofluorescence study, we found that astrocytes located immediately adjacent to the lesion site expressed high levels of C/EBPδ but low levels of RhoA in wild-type mice after SCI. From the aspect of neuroprotection, C/EBPδ, under such conditions, appears to immobilize these astrocytes to seclude the inflammatory cells in the lesion. Nevertheless, this benefit, if there is any, is apparently outweighed by the detrimental glial scar that adversely affects motor function recovery, as observed in the wild-type mice with SCI. On the other hand, injured *C*/*EBPδ* ^−/−^ mice show reduced glial scarring and improved locomotor recovery. In fact, we have demonstrated previously that activated microglia/macrophages are reduced in the brain of amyloid precursor protein transgenic and *C*/*EBPδ*-deficient (*APP*Tg/*C*/*EBPδ* ^−/−^) mice, indicating a relatively mild inflammation in these mice due to the lack of Cepbd compared with the wild-type mice [45].

We found that C/EBPδ directly repressed the transcription of RhoA, but not Cdc42, Rac1, or FAK in IL-1β-treated wild-type astrocytes. Sumoylation, a post-translational modification process affecting the structure and subcellular localization of a protein, is required for the suppression of C/EBPδ-dependent transcription in cells treated with EGF and lipogenic inducers [46, 47]. However, whether this post-translational modification participates in the C/EBPδ-mediated repression of RhoA transcription in IL-1β-treated astrocytes needs to be further investigated.

Our research suggested that C/EBPδ inhibited astrocyte migration at the lesion border but, contradictorily, C/EBPδ also enhanced the overall glial scarring in wild-type mice after SCI. This finding raises an intriguing question about whether C/EBPδ concomitantly induces promoting factors that predominantly counteract the inhibitory effect of C/EBPδ on RhoA expression, leading to enhanced overall motility of astrocytes and thus extensive glial scar formation. Indeed, we found that IL-1β-treated astrocytes with increased C/EBPδ expression produced MMP-3 in the conditioned medium, which facilitated the migration of non-IL-1β-treated, inactive astrocytes in vitro.

In the injured wild-type mouse, moreover, astrocytes exhibited relatively low C/EBPδ but high RhoA expression in the penumbral region lying between the lesion border and the less injured tissue where MMP-3 was evident, suggesting higher migratory activity of these astrocytes that ultimately form a glial scar. Conversely, the glial scarring was not apparent in the *C*/*EBPδ* ^−/−^ mouse, despite obvious RhoA expression, because of low MMP-3 levels in the injured cord. This outcome supports the involvement of astrocytic C/EBPδ in MMP-3 expression, which promotes astrocyte motility after SCI.

MMP-3 is upregulated in astrocytes after brain injury. Our previous micro-array data showed that C/EBPδ upregulates MMP-3 expression in human glioblastoma-astrocytoma U373MG cells. Moreover, MMP-3 may activate the expression of MMP-9 in tumor cells [48]. MMP-9 has been demonstrated to facilitate astrocyte migration, promote glial scar formation, and inhibit axonal regeneration and functional recovery after SCI [12, 49]. Our results add to further understanding of how C/EBPδ contributes to astrocyte motility and glial scar formation through upregulation of MMPs after SCI.

In addition to astrocytes, microglia also express MMP-3, for instance, upon amyloid-β stimulation [50]. Although previous studies suggest that IL-1β has no detectable effect on the expression of MMP3 gene in pure microglia culture [51], the involvement of microglia in the process of astrocyte migration and glial scar formation has to be taken into account when microglia-astrocyte interaction exists. It is noteworthy that the astrocyte culture system used in the present research is mixed with microglia at least to a small extent. Thus the possibility that microglia may affect the migratory behavior of astrocytes in vitro cannot be completely excluded unless microglia-free astrocyte cultures are used [51, 52]. Moreover, although we demonstrated that MMP-3 was expressed in astrocyte cultures by IL-1β stimulation in vitro, whether astrocytic expression of MMP-3 was solely IL-1β-dependent in the injured spinal cord needs further investigations. We showed previously that elevated expression of C/EBPδ in astrocytes indirectly promotes the activation of microglia through the expression of a chemoattractant monocyte chemotactic protein-1 (MCP-1) [26]. Nevertheless, additional research on the interplay between astrocytes and microglia upon the influence of C/EBPδ will better elucidate the role of microglia in glial scar formation during wound healing after SCI.

As discussed above, inflammatory responses following the injury are triggers for glial scar formation [53]. C/EBPδ has been suggested to regulate many inflammatory molecules, such as TNF-α, IL-1β, IL-6, CXCL1, IL-17A, MCP-1, PTX3, and COX-2 [45, 46, 54, 55]. In addition to C/EBPδ, activation of transcription factors NF-κB, CREB, and STAT3 in astrocytes has been observed in many neuro-inflammatory diseases. Several studies have shown that NF-κB, CREB, and STAT3 are the upstream transcriptional activators of the C/EBPδ gene in various cell types [56–58]. In astrocytes, CREB has been suggested to play an important role in C/EBPδ transcription [59]. Among these three transcription factors, the activation of astrocytic NF-κB and STAT3 has been suggested to play important roles particularly in the upregulation of intermediate filaments, hypertrophy of the cell body, and glial scar formation after SCI [29, 60, 61]. However, the involvement of astrocytic CREB in SCI remains unclear. More detailed studies regarding the transcriptional activation of C/EBPδ in response to IL-1β in astrocytes are needed to further define the roles of C/EBPδ, NF-κB, CREB, and STAT3 in reactive astrogliosis after SCI.

In conclusion, our studies demonstrate for the first time the involvement of C/EBPδ in glial scar formation by modulating astrocyte motility after SCI. Although inflammation is a series of complex processes initiated from the original insult, inhibition of C/EBPδ or C/EBPδ-mediated downstream genes or proteins may ultimately become a therapeutic option in the future to reduce glial scarring and associated secondary pathogenesis to improve functional outcomes after SCI.

## Materials and Methods

This study was approved by the Institutional Animal Care and Use Committee at National Cheng-Kung University, Taiwan, in accordance with the Guideline for the Care and Use of Laboratory Animals. The *C*/*EBPδ* ^−/−^ mice were provided as a gift by Dr. E. Sterneck [62].

### Primary Cultures of Mouse Astrocytes

Primary astrocytes were isolated from the cerebral cortex of wild-type or *C*/*EBPδ* ^−/−^ newborn mice by mechanical dissociation. The isolated cells were then filtered through a 70-μm nylon strainer (Millipore, Bedford, MA) and cultured in a poly-l-lysine-coated flask containing the medium as previously described [12, 24, 29]. This method of cortical astrocyte culture is widely used in research on spinal cord injury in vitro. The purity of astrocyte cultures was approximately 95 %, determined by anti-GFAP immunostaining and nuclear staining with Hoechst dye.

### Scratch Wound Assay

The mouse primary astrocytes were grown in Dulbecco’s modified Eagle’s medium (DMEM) with 10 % Fetal bovine serum (FBS) for 24 h. Then, the confluent monolayer of astrocytes was scratched with a Culture-Insert (ibidi GmbH, Martinsried, Germany). After washing with phosphate-buffered saline (PBS), the experimental cells were grown in 10 ng/mL IL-1β or in the conditioned media described above. The scratched area was photographed at 0 and 21 h after the scratch wound was made for subsequent quantitative analyses using NIH ImageJ image processing software. The size of the scratched area covered by migrating astrocytes at 21 h was first delineated, measured, and then compared with the measurement obtained at 0 h (100 %).

### Contusive Spinal Cord Injury

Adult female wild-type or *C*/*EBPδ* ^−/−^ mice with the C57BL/6 genetic background, weighing 20–30 g and 3 ∼ 6 months of age, were anesthetized with 2.5 % Avertin (0.02 mL/g body weight, intraperitoneal administration, tribromoethanol; Sigma, St. Louis, MO) and subjected to a moderate contusion injury to the spinal cord at the mid-thoracic level as described previously [49]. Briefly, a laminectomy was performed at the eighth thoracic vertebra and a 2-g weight was dropped from a height of 5 cm onto the exposed dura mater. After the injury, the overlying muscles were sutured, and the skin was closed with wound clips. The body temperature of the animals was maintained at 37 °C with a heating pad throughout the surgery and during the recovery from anesthesia. Postoperative care included subcutaneous administration of antibiotics and manual expression of the bladder twice per day.

### Assessment of Open-Field Locomotion

The 9-point Basso Mouse Scale (BMS) for locomotor rating [63] was used to examine the locomotor recovery of the injured animals. This rating scale assessed limb movement, stepping, coordination, and trunk stability in an open field (53 × 108 × 5.5 cm). Injured animals with better locomotor recovery scored higher. One trial, which lasted for 3 min, was performed before the injury (day 0) and on day 7, 14, 21, and 28 after the injury.

### Rotarod and Footprint Analysis

The rotarod test was conducted as described previously [61]. Briefly, the injured mice were placed on a rod rotating from 0 to 30 rpm, and the time that the mice ran on the rod before falling was measured. Each trial lasted for a maximum of 3 min and was repeated three times. For the footprint analysis, the hindpaws of the mice were painted with ink to record the walking pattern during continuous locomotion across a paper runway (3 × 30 cm) 4 weeks after the injury. The stride lengths were measured and analyzed only when the mice ran at a constant velocity. Strides over the first and last 5 cm of the passage were excluded because of the variation in the walking velocity of the mice.

### Quantitative PCR

Total RNA was extracted using the TRIsure RNA extraction reagent (Invitrogen). cDNA synthesis was performed with an RT reaction using SuperScript III (Invitrogen). Quantitative PCR (qPCR) was conducted using KAPA SYBR FAST qPCR Master Mix (Life Technologies Corporation and Kapa Biosystems Inc.). PCR was conducted using a CFX connect Real-Time PCR System (BIO-RAD) with the following pairs of specific primers: mouse C/EBPδ (forward): 5′-CTCCCGCACACAACATACTG-3′ and C/EBPδ (reverse): 5′-AGTCATGCTTTCCCGTGTTC-3′, mouse MMP-3 (forward): 5′-TGGAACCTGAGACATCACCA-3′ and MMP-3 (reverse): 5′-GATGGAAGAGATGGCCAAAA-3′, mouse RhoA (forward): 5′-TGGTTGGGAACAAGAAGGAC-3′ and RhoA (reverse): 5′-CAAGATGAGGCACCC AGACT-3′. Then the quantity of mRNA was determined by NIH ImageJ software. The result of each group was normalized with that of the IL-1β-untreated wild-type astrocytes and was expressed as the difference in folds.

### Western Blot Analysis

The cells were harvested and lysed with modified RIPA buffer (50 mM Tris-HCl [pH 7.4], 150 mM sodium chloride, 1 mM ethylenediamine tetra-acetic acid, 1 % NP40, 0.25 % sodium deoxycholate, 1 mM dithiothreitol, 1 mM PMSF, 1 μg/mL aprotinin, and 1 μg/mL leupeptin). The lysates were resolved on a sodium dodecyl sulfate gel containing 10 % polyacrylamide, then transferred to a polyvinylidene difluoride nylon membrane and probed with primary antibodies for the target proteins at 4 °C overnight. The primary antibodies used included anti-p84 (Clone: 5E10, GeneTex), −Rac1 (GTX100761, GeneTex), −Cdc42 (GTX100904, GeneTex), −RhoA (ab68826, Abcam, Cambridge, MA), −FAK (#3285, Cell Signaling Technology, Danvers, MA), −MMP-3 (JM-3523-100, MBL International, Woburn, MA), and -β-Actin (sc-1616, Santa Cruz Biotechnology, Santa Cruz, CA). As the molecular weight of MMP-3 is close to that of β-actin, p84 (84 kDa) was used as the loading control for probing MMP-3. The specific proteins were detected by incubation with a peroxidase-conjugated secondary antibody at room temperature for 1 h. The signals were visualized using an enhanced chemiluminescence Western blot system (Pierce, Rockford, IL). Then the chemiluminescence intensity was quantified using NIH ImageJ software. The result of each group was normalized with that of the untreated wild-type astrocytes and was expressed as the difference in folds.

#### Luciferase Reporter Assay

The 5′ flanking regions of the *MMP*-*3* genes were obtained by PCR with genomic DNA and then individually cloned into a pGL3 basic vector. The primers for PCR of the genomic DNA were as follows: MMP-3 (forward): 5′-XhoI-CCGCTCGAGCGGAAGACTGGAGAAGGAGGCTG-3′ and MMP-3 (reverse): 5′-HindIII-CCCAAGCTTGGGCTGCCTCCTTCTAGGTCCAC-3′ and RhoA (forward): 5′-KpnI-GGGGTACCCCCAGGAGAACCCAATGGTACAG-3′ and RhoA (reverse): 5′-XhoI-CCGCTCGAGCGGATCCACGCCCTGAGAGCTAGAC-3′. For the reporter assay, the cells were transfected with the reporters and expression vectors as indicted using poly-Jet (SignaGen, Ijamsville, MD). The lysates of the transfected cells were harvested following the manufacturer’s instructions for the luciferase assay. The luciferase activity of the reporters in each cell group was quantified and normalized with that of the IL-1β-untreated wild-type astrocytes.

### Chromatin Immunoprecipitation Assay

Briefly, mouse primary astrocytes were treated with 1 % formaldehyde for 15 min. The cross-linked chromatin was then prepared and sonicated to an average size of 500 base pairs. The DNA fragments were immunoprecipitated with a specific antibody recognizing C/EBPδ (sc-636, Santa Cruz Biotechnology) or a rabbit immunoglobulin (Ig) G as the control at 4 °C for 12–16 h. After reversal of the cross-link between the proteins and the genomic DNA, the precipitated DNA was amplified by PCR with primers related to the specific regions of the genomic loci of the target genes. The primers were as follows: MMP-3 (forward), 5′-CAAACATTACAGCTCTGGAAGG-3′ and MMP-3 (reverse), 5′-CTTAAGCCCAACTTTTATAGAGTGG-3′ or RhoA (forward), 5′-CAGGAGAACCCAATGGTACAGT-3′ and RhoA (reverse), 5′-GCAAGCGAAGTAGATCTTCC-3′.

### Immunofluorescence Analysis

Frozen sections of the spinal cord were cut transversely at 20 μm, mounted onto glass slides, and treated with protein blocker/antibody diluents (Bio SB, Santa Barbara, CA, USA) for 1 h. Then, the sections were incubated overnight with primary antibodies diluted in the same buffer at 4 °C for immunofluorescent staining. The primary antibodies included anti-GFAP (Clone: 2.2B10, Invitrogen), −C/EBP*δ* (Geneka Biotechnology, Montreal, Canada), −Ki-67 (M3064, Spring Bioscience, Pleasanton, CA), −RhoA, and -MMP-3 antibodies. For the staining of cell cultures, primary astrocytes were post-fixed in 4 % paraformaldehyde in PBS for 20 min, followed by 70 % methanol in PBS at −20 °C for 10 min. The fixed primary astrocytes were further incubated with primary antibodies against the target proteins in 3 % BSA at 4 °C. Frozen section or primary astrocytes were then washed with 0.2 % Triton X-100 in PBS, incubated with Alexa 488- or 555-conjugated secondary antibodies (Invitrogen) for 1 h at room temperature, and washed again with 0.2 % Triton X-100 in PBS. The glass slides were counter-stained and cover-slipped with ProLong Gold anti-fade reagent with 4′,6-diamidino-2-phenylindole for immunofluorescence microscopy. The intensity of immunoreactivity was analyzed quantitatively using TissueQuest imaging software (TissueGnostics, Austria) with a module in which a threshold value was first determined empirically to eliminate the background staining of the uninjured cord tissue and then applied to the sections containing the lesion epicenter. The area with fluorescent intensity higher than the threshold value was considered immunoreactive and was calculated, averaged with those of the same mouse group, and reported as a percentage. Co-expression or co-localization of GFAP and C/EBPδ immunoreactivity was calculated by the formula [(GFAP/ C/EBPδ double positive signals)/ total C/EBPδ positive signals]%.

### Luxol Fast Blue Assay

Luxol Fast Blue staining was used to visualize the residual white matter around the lesion epicenter of the injured spinal cord. Cross sections of the spinal cord were rinsed in 50 % ethanol, followed by gradient dehydration in 75, 85, and 95 % ethanol for 3 min each. The sections were stained with 0.1 % Luxol Fast Blue containing 0.5 % glacial acetic acid in 95 % ethanol at 60 °C for 3 h. After a rinse in 95 % alcohol and distilled water, the sections were incubated in 0.05 % lithium carbonate solution for 3 min, followed by color differentiation in 70 % ethanol and distilled water. The sections were dehydrated and clarified in gradient ethanols and xylene, respectively, and cover-slipped with Micromount. The area of the residual white matter was then measured by the NIH ImageJ software.

### Lentiviral Knockdown

The virus was produced from Phoenix cells by co-transfecting various shRNA expression vectors in combination with the pMD2.G and psPAX2 expression vectors. The expression vectors were obtained from the National RNAi Core Facility located at the Genomic Research Center of the Institute of Molecular Biology, Academia Sinica, Taiwan. After determining the viral infection efficiency, the lentivirus containing shβ-galactosidase (shLacZ) or shMMP-3 was used to infect primary astrocytes for 48 h. The shRNA sequences in the lentiviral expression vectors were shLacZ: 5′-CCGGTGTTCGCATTATCCGAACCATCTCGAGATGGTTCGGAT AATGCGAACATTTTTG-3′, and shMMP-3: 5′-CCGGCC CACATATTGAAGAGCAATACTCGAGTATTGCTCTTCAATATGTGGGTTTTTG-3′.

### Preparation of Conditioned Media

Conditioned media were harvested from primary cultures of wild-type or *C*/*EBPδ* ^−/−^ astrocytes infected with the shLacZ or shMMP-3 lentivirus. Briefly, experimental cells were grown in DMEM with 10 % FBS for 24 h and then treated with or without IL-1β for 6 h in serum-free DMEM. After washing with PBS, the IL-1β-pretreated cells were cultured in serum-free DMEM. After another 12 h, the supernatants from the cultures were centrifuged, filtered, and stored at −80 °C for further use.

### Statistical Analysis

All experiments were repeated at least three times, and the data were analyzed for statistical significance using the two-tailed unpaired Student’s *t* test (Prism 5 software). Two-way ANOVA, followed by Bonferroni’s post hoc test, was performed for multiple comparisons between groups. The data were expressed as the means and standard error of mean (±SEM). A statistically significant difference was defined at *p < 0.05, **p < 0.01, and ***p < 0.001.

## Electronic Supplementary Material

Below is the link to the electronic supplementary material.

ESM 1 (PPTX 129 bytes)
