# Supplementary material for: Astrocytic CCAAT/Enhancer-Binding Protein Delta Contributes to Glial Scar Formation and Impairs Functional Recovery After Spinal Cord Injury
Source: Mol Neurobiol. 2015 Oct 28;53(9):5912–27. doi: 10.1007/s12035-015-9486-6 (PMC5085997; doi:10.1007/s12035-015-9486-6)
Supplement: Supplementary file 1 — (PPTX 129 bytes) [file 12035_2015_9486_MOESM1_ESM.pptx]

## Slide 1
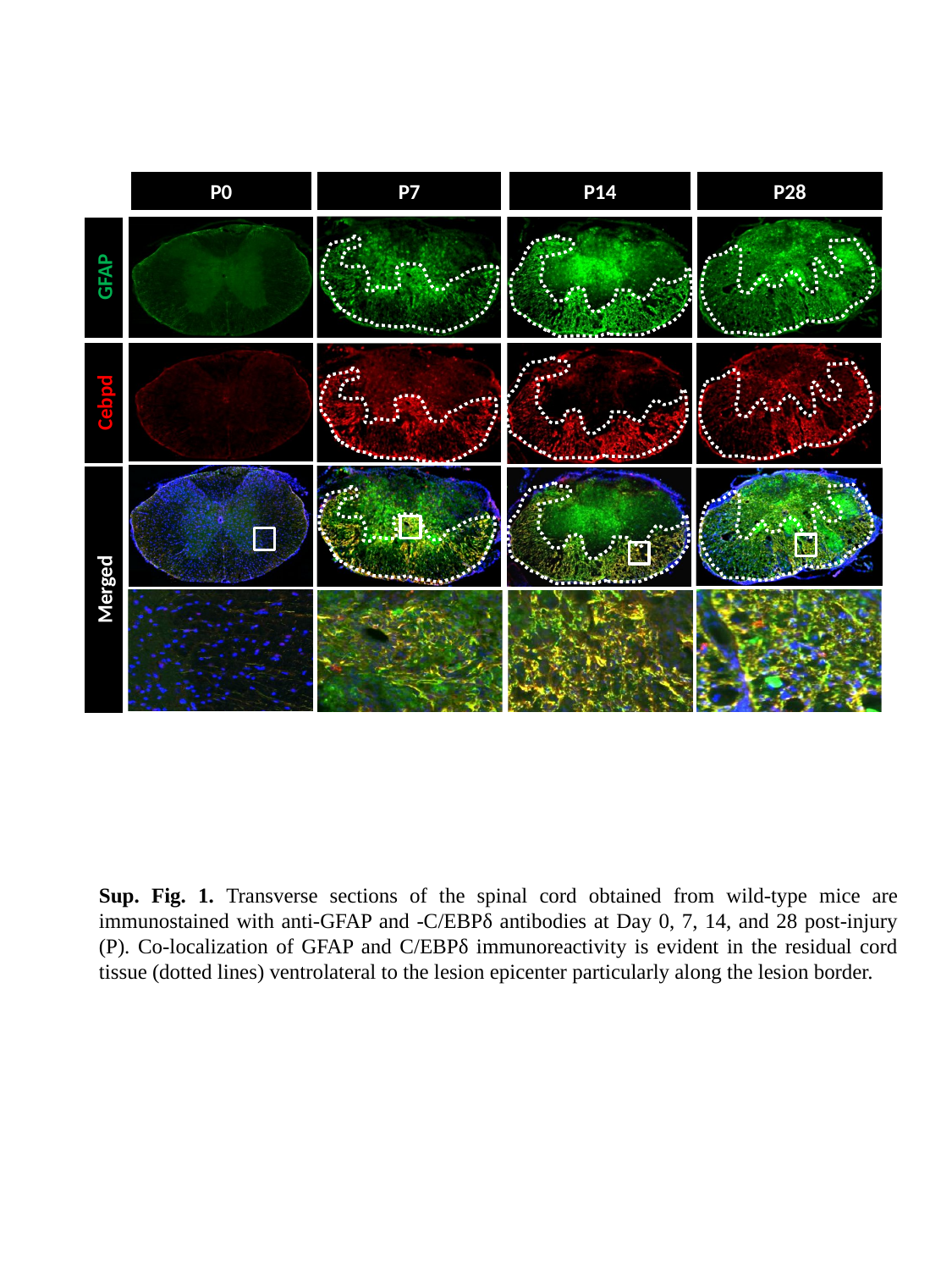

P0
P7
P14
P28
GFAP
Cebpd
Merged
Sup. Fig. 1. Transverse sections of the spinal cord obtained from wild-type mice are immunostained with anti-GFAP and -C/EBPδ antibodies at Day 0, 7, 14, and 28 post-injury (P). Co-localization of GFAP and C/EBPδ immunoreactivity is evident in the residual cord tissue (dotted lines) ventrolateral to the lesion epicenter particularly along the lesion border.

## Slide 2
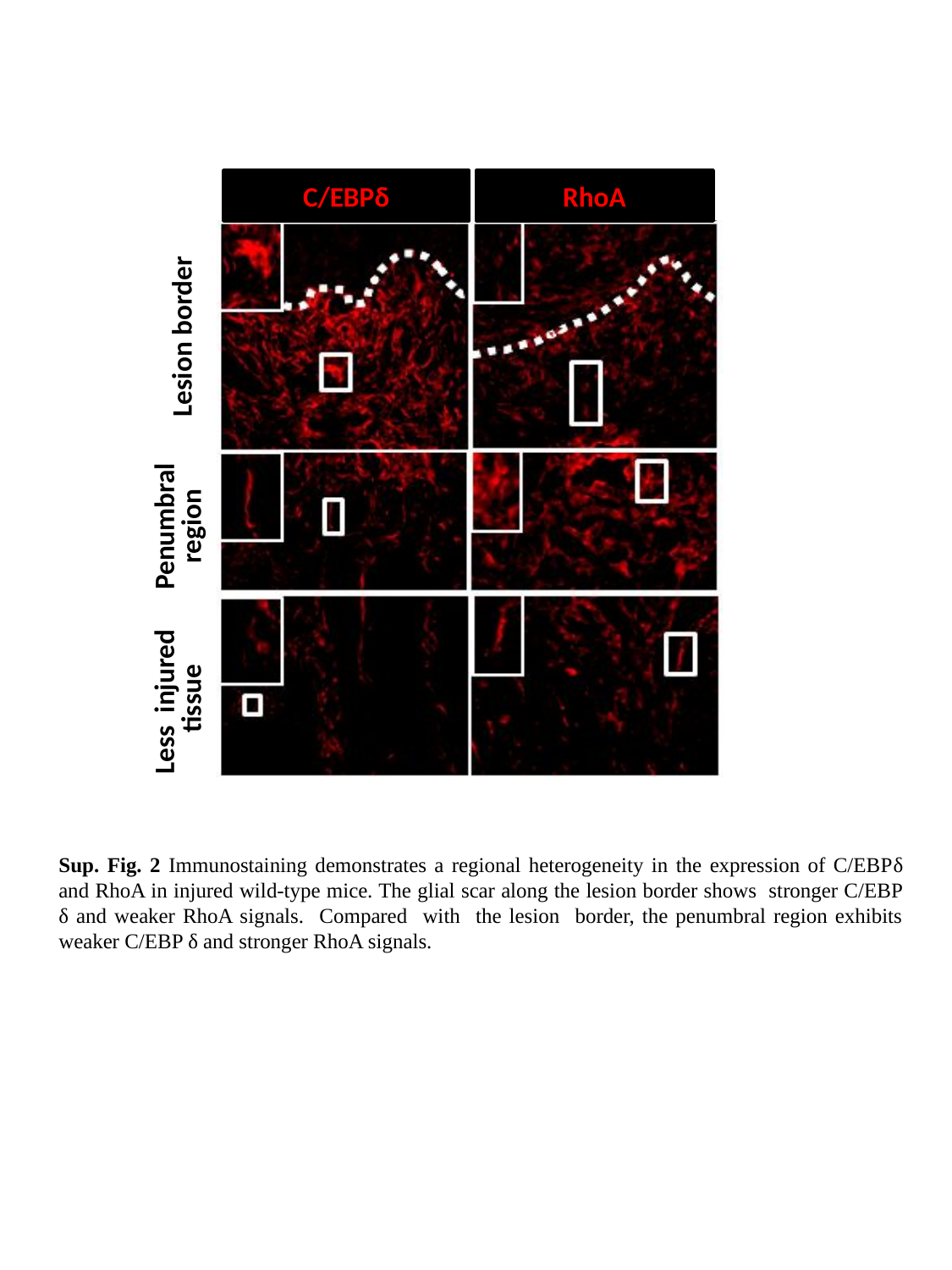

C/EBPδ
RhoA
Lesion border
Penumbral
region
Less injured
tissue
Sup. Fig. 2 Immunostaining demonstrates a regional heterogeneity in the expression of C/EBPδ and RhoA in injured wild-type mice. The glial scar along the lesion border shows stronger C/EBP δ and weaker RhoA signals. Compared with the lesion border, the penumbral region exhibits weaker C/EBP δ and stronger RhoA signals.
